# A Review of Contact Lens-Induced Limbal Stem Cell Deficiency

**DOI:** 10.3390/biology12121490

**Published:** 2023-12-05

**Authors:** Yhu Fhei Lee, Dayna Wei Wei Yong, Ray Manotosh

**Affiliations:** 1 Yong Loo Lin School of Medicine, National University of Singapore, Singapore 117597, Singapore; 2 Department of Ophthalmology, National University Hospital, Singapore 119074, Singapore; 3 Department of Ophthalmology, Yong Loo Lin School of Medicine, National University of Singapore, Singapore 119228, Singapore

**Keywords:** limbal epithelial stem cells, LESC transplantation, contact lens, limbal stem cells, limbal stem cell deficiency

## Abstract

**Simple Summary:**

Contact lens (CL)-induced limbal stem cell deficiency (LSCD) is not well-known and is one of the lesser-understood causes of LSCD. With increasing contact lens usage worldwide, its prevalence is bound to increase. This review will critically discuss the underlying risk factors, methods of diagnosis and treatment of CL-induced LSCD.

**Abstract:**

Limbal stem cell deficiency (LSCD) is a pathologic condition caused by the dysfunction and destruction of stem cells, stem cell precursors and limbal cell niche in the corneal epithelium, leading to severe conjunctivalization of the cornea. Etiologies for LSCD span from congenital (aniridia), traumatic (chemical or thermal injuries), autoimmune (Stevens–Johnson syndrome) and iatrogenic disease to contact lens (CL) wear. Of these, CL wear is the least understood and is often a subclinical cause of LSCD. Even with recent advances in LSCD research, limitations persist in establishing the pathogenesis and treatment guidelines for CL-induced LSCD. A literature search was conducted to include original articles containing patients with CL-induced LSCD. This review will critically discuss the complex pathophysiology behind CL-induced LSCD, the underlying risk factors and epidemiology of the disease as well as methods to obtain a diagnosis. Various treatment options will be reviewed based on proposed treatment strategies.

## 1. Introduction

On the ocular surface, the homeostasis of the corneal epithelium is constantly maintained by the limbal stem cells within the basal epithelial layer of the limbus [1,2,3,4,5]. Within the limbal niche and its microenvironment, complex signaling pathways mediate cell-to-cell interactions that promote wound healing [2,3,4] and ensure adequate corneal epithelial cell turnover [6,7]. When these cellular processes are disrupted, there may be clinical manifestations that may result in severe ocular complications [2,3,4].

In limbal stem cell deficiency (LSCD), the limbal stem cell reserve is depleted and/or becomes dysfunctional, leading to a loss of corneal epithelial replenishment [2,4,7,8]. Recent advances in limbal stem cell transplantation have improved the surgical approach to differing severities of LSCD [1,2]. Therapy for LSCD was greatly cemented and solidified in 2019 with the Global Consensus on Definition, Classification, Diagnosis, Staging and Management of LSCD [9].

There are a multitude of etiologies for LSCD (see Table 1), spanning congenital, traumatic, autoimmune and idiopathic, with contact lens wear being one of the lesser understood and often subclinical traumatic causes of LSCD [2,5,8].

In the past, CL use has led to other corneal pathologies such as CL-induced keratopathy, superior limbic keratoconjunctivitis, infectious keratitis, etc. [8]. For eye wear with extensively documented complications, studies are still limited in understanding pathogenesis and standardizing treatment for CL-induced LSCD. This review will provide an overview of the epidemiology, risk factors, diagnosis, pathophysiology, treatment options and current gaps in our understanding of CL-related LSCD.

## 2. Epidemiology

There are an estimated 125 million CL wearers worldwide [2,15], with an estimated 6% annual increase based on sales growth [16,17]. The prevalence of CL use by individuals between the ages of 15 and 25 is 40.5% [16]. In the United States, there are an estimated 40.9 million U.S. adult CL users. Around 90–93% of CL users have been reported to use soft CL [18,19]. Out of 1000 respondents in the Contact Lens Risk Survey, approximately 99% of CL users had at least one CL-related hygiene behavior that has associated risks for eye infection or inflammation [18]. This included sleeping overnight in CL (50.2%) and not fully replacing disinfecting solutions (55.1%) [18].

The most common uses for CL include optical and cosmetic purposes [20]. Other indications include therapeutic (i.e., corneal pathologies, dry eye), diagnostic (i.e., fundus photography, electroretinography, biometry for severely disorganized cornea), preventive (i.e., exposure keratitis, myopia progression) and occupational (i.e., pilots, police, athletes) [20]. A survey of 499 Korean CL users reported that the age of starting CL use was decreasing and the use of cosmetic CL was increasing [21]. The mean age of starting CL use was reported at 24.9 years [21]. The mean overnight wearing days (per month) was noted to be 8.4 days for all subjects: 9.7 days for soft CL, 5.3 days for cosmetic CL, and 16 days for rigid gas permeable CL [21].

Globally, 59–75% of LSCD cases are female [5,22]. Across 45 million CL users in the United States, approximately 2.4–5% progress to LSCD [23,24]. Out of all LSCD cases, it is noted that 15% of cases are secondary to CL use [12,25]. In a study by Martin et al., out of 2.4% of soft CL wearers with focal LSCD findings, only 28.6% of CL wearers reported subjective disease-related symptoms [23]. The reported incidence of LSCD cases may be severely underestimated, with the majority of milder asymptomatic cases possibly being unreported as they are asymptomatic [11,23]. Incidences of CL-focal LSCD have also increased in recent years for undetermined reasons [22].

In LSCD cases, the average CL use was 14.2–17.6 years and 12.5–16.25 h per day. However, patients could manifest LSCD as early as 6–12 months of use [8,11,12,23,24,26]. The mean age at diagnosis was 36.9 years (32–42 years) [11,23,24]. While it may be expected that the majority of CL-induced LSCD cases should affect both eyes, only half of these cases had bilateral clinical involvement [11]. A total of 58% of CL-induced LSCD cases had a history of ocular disorders and co-morbidity [11].

Almost all reported patients with CL-induced LSCD were soft CL users [10,11,23,27,28]. Some cases were related to rigid gas permeable lens wear [29], but no risk or complications have been associated with hybrid or scleral lens use as of yet [8]. In comparing lens-wearing schedules, both daily-wear and extended-wear use notably increase LSCD incidence without much difference [10,23,28,29].

## 3. Pathophysiology

What has remained uncertain is how CL-induced LSCD transitions from a reversible subclinical form to severe LSCD with irreversible corneal damage. A two-hit hypothesis has been proposed where, firstly, the LSCs are slowly stressed and become dysfunctional over a chronic course of soft CL wear. At this point, the LSCs are functionally impaired but still subclinical. Eventually, a “second hit”, which can be secondary to severe lid disease, chronic ocular surface inflammation or other underlying disease processes, acts as a tipping point. The remaining viable functions of the LSCs are fully exhausted and then progress to severe LSCD [24,30]. Further study is needed to better understand how this hypothesis specifically correlates to CL-induced LSCD pathogenesis.

CL-induced LSCD pathogenesis is generally accepted to be multifactorial and complex and has not yet been fully defined in showing a clear causal relationship between CL wear and LSCD. These pathological factors are largely classified into mechanical trauma, effects of dry eye on the ocular surface, toxicity effects of preservatives in lens cleaning solutions and hypoxia.

Tears provide lubrication to the corneal surface, preserving its function by supplying mucin and antimicrobial factors and replenishing oxygen and nutrients [31]. Tear film stability and CL interaction with the corneal surface rely on each other to maintain corneal stability [19]. While CL usually coexist with the tear film without complications, they sometimes disrupt the tear film and impair tear exchange. In such cases, the CL–tear film interaction increases tear film osmolarity and alters tear film composition as a thinner and unstable lipid layer with delayed spread is formed [19]. Furthermore, less tear film additionally traumatizes the CL [19]. Given the multifactorial nature of CL characteristics, the exact nature of how CL affect the tear film is challenging to fully comprehend [8].

There are other factors that affect the tear film, leading to dry eye [19]:

- Lens diameter, blinking;
- Volume and rate at which tears are produced/secreted;
- CL properties: lens-to-cornea fitting relationship, material composition, design (base curve/diameter combination, zone size, etc.).

Preservatives are constantly used for disinfecting and cleaning CL. Previous studies have shown that the combined use of a multipurpose solution and a silicone hydrogel lens has led to epithelial cell damage [32,33,34]. With prolonged use, preservatives in multipurpose solutions can produce hypersensitivity reactions or epithelial disturbance either from direct toxic effects or by inducing secondary inflammation [15,26,35,36]. However, solutions with the same percentage of preservatives can produce different straining patterns due to other ingredients (chelating and wetting agents, etc.) and their interactions with various lens properties [37,38]. Currently, modern preservatives are generally in use due to their relatively low levels of ocular toxicity. This includes Polyquaternium-1 (PQ-1), biguanides (PHMB) and Aldox (Myristamine-Propyl-Dimethylamine 0.0005%). Preservative-free solutions include Hydrogen peroxide and Povidone-iodine. Older-generation preservatives that were previously used are Thimerosal and Benzalkonium chloride (BAK). Modern preservatives have a higher molecular weight, which reduces the risk of uptake and release from CL, minimizing risk to the ocular surface, whereas older-generation preservatives have been widely documented to have significant ocular toxicity effects [39,40].

Thimerosal toxicity has been widely presumed to cause LSCD [8]. It has been shown to induce immediate toxic responses, both in vitro [41] and in vivo [42], via the inhibition of mitotic activity in human corneal epithelial cells. The small molecular size of Thimerosal allows it to be easily absorbed and released by CL [39]. It then leaches from the CL onto the ocular surface, leading to corneal irritation, limbal epithelial changes and corneal staining after CL wear [39]. Thimerosal toxicity has also been known to cause corneal squamous metaplasia, differentiating it as a separate pathological condition from a normal CL-induced LSCD case [15]. Due to its known toxicity, thimerosal has not been in use since the 1980s [15].

BAK (Benzalkonium chloride) is known to affect the corneal epithelium and limbal stem cell environment, which could potentially progress to LSCD [43]. BAK is not used in CL multipurpose solutions due to its easy absorbability and uptake by CL and its well-documented history of cytotoxicity [43]. PHMB (Polyhexamethylene biguanide) preservatives have been shown to have more corneal staining than hydrogen peroxide-based solutions [37]. Many in vivo studies have also reported higher-grade corneal staining with PHMB compared to PQ-1 when wearing both hydrogel [37] and silicone hydrogel CL (the worse being silicone hydrogel CL) [32,37,44,45,46,47]. In other studies, H202 was found to be comparatively better than PHMB in improving patient symptoms after 90 days of use [48,49]. However, with many conflicting studies on their comparative ocular toxicity, PQ-1 and PHMB are considered to have a generally similar efficacy and safety profile [40]. Being preservative-free, P-I and H_2_O_2_ have excellent safety profiles and are reported to have less ocular toxicity than modern preservative solutions, making them an alternative to switch to if patients are experiencing preservative toxicity [40].

Tanti et al. ranked CL solution toxicities in the following descending order: Alcon Optifree Express (PQ-1 0.0001%, Aldox 0.0005%), Bausch and Lomb Re-Nu MultiPlus (PHMB 0.0001%), Complete Solution by Abbott Medical Optics (PHMB 0.0001%) [40,50]. However, further research needs to be conducted to assess how the release of compounds is mediated by different lens types and how different physical properties and surface treatments affect the preferential adsorption/release profile of compounds by silicone hydrogel lenses [50].

Trauma plays an essential role in CL-induced LSCD. CL normally move against the ocular surface with a movement of 0.1–0.4 mm, which is considered comfortable for CL users. When one blinks, tears are dispelled from the interface between the lens and the ocular surface, inducing friction between the lens and the eye [51]. Moreover, when lens movement becomes inadequate, the tear film behind the lens decreases, inducing more friction [51]. Areas of high pressure, like the superior limbus, in particular, and the inferior limbus, are especially affected by lens movement during blinking, possibly leading to chronic trauma [52]. Differences in eye and eyelid anatomy, such as narrower palpebral apertures, which are more common in Asians, may contribute to increased mechanical trauma from contact lens use [52].

Hypoxia results from physical barriers created by CL that prevent oxygen delivery to the cornea [35,53]. In the tear films of CL users, raised levels of lactate dehydrogenase have been noted, which further supports CLs’ hypoxic effects [54]. Hypoxia causes limbal stress and inflammation, which detriments the limbal niche, contributing to CL-induced LSCD [10,24,27]. It is usually insufficient for hypoxia to be the sole causative factor of LSCD; hence, it is the cumulative hypoxic effect with other pathological factors (mechanical trauma, lens preservative toxicity) that result in clinical LSCD [11,23,55]. Superior limbal localization of LSCD can also be explained by increased hypoxia detected in the superior limbus [12]. It has also been theorized that the increased peripheral thickness in high negative lenses could worsen hypoxia on the limbal surface [56].

## 4. Risk Factors

Many studies have attempted to elicit risk factors that show an association with CL-induced LSCD. While many factors have been well established with past research and other associated ocular diseases, some remain unproven and hypothetical.

### 4.1. Contact Lens Properties

The type of contact lens used has been shown to affect the incidence of CL-induced LSCD. CL characteristics that increase mechanical trauma are less flexible material (i.e., silicone hydrogel compared to hydrogel), bulky lenses, lenses with steepened base curves and poorly fitted lenses [23,55,57]. These characteristics also correlate clinically with the type of contact lens patients use (silicone hydrogel, hybrid lens, rigid lens, mini-scleral/scleral lens). A possible explanation is that rigid lenses are smaller in diameter (5–6 mm) and more mobile than soft lenses [49,58]. Soft lenses usually have an on-eye displacement during blinking of 0.3–0.5 mm, whereas rigid lenses displace by about 1 to 2 mm [49,58]. The increased material rigidity and smaller diameter allow more lens movement with every blink, which increases tear exchange under the lens, making RGP lenses less likely to cause LSCD [59,60].

Compared to hydrogel lenses, silicone hydrogel lenses are noted to be superior in oxygen permeability and other aspects [22]. However, certain materials in silicone hydrogel lenses could absorb components of multipurpose solutions during overnight disinfection, gradually releasing them onto the cornea throughout the day [22,32,33,34].

Tanti et al. demonstrated in-vitro toxicity to human corneal epithelial cells in cell culture by multipurpose lens cleaning solutions released from silicone hydrogel lenses [50]. Conversely, therapeutic scleral lenses may have a role in treating LSCD and preventing adverse effects on the limbus [11].

The Prosthetic Replacement of the Ocular Surface Ecosystem (PROSE) lens is a rigid gas-permeable scleral lens that completely covers the limbus and cornea, creating a fluid reservoir that hydrates and sustains LSCs and the limbal niche and guards them from trauma [28,61,62]. Studies indicate visual improvement and reversal of LSCD, making it a viable option for mild to moderate LSCD and potentially sparing severe LSCD patients from surgical or medical intervention [10,62]. However, there remains a gap in research regarding whether particular lens types (single vision, toric, multifocal) or lens power correlate with increased mechanical trauma [8].

### 4.2. Factors Causing Dry Eye

Tears help to nourish, lubricate and protect the corneal surface [31]. Without an adequate tear film surface, it may lead to dry eye, which could result in a multitude of corneal pathologies. Hence, risk factors affecting the tear film have been identified that may theoretically predispose to CL-induced LSCD. Anatomical differences in eyelid structure and corneal surface could increase mechanical trauma and, subsequently, the incidence of LSCD [57].

Due to such differences, when compared to non-Asians, Asian CL users have reportedly higher incidences of dry eye, symptoms like ocular discomfort and increased corneal staining [55,63]. It has been postulated that the Asian population possibly has dryer eyes and decreased tear films due to physiological differences [55,63]. Nevertheless, Termote et al. found no indication of a racial predisposition to focal LSCD [22]. Steeper corneas typically exhibit less CL movement, which potentially reduces chronic trauma but also reduces tear film renewal [22]. Tighter lids with narrower palpebral apertures could intensify CL movement against a less steep cornea, exacerbating the dry eye effect on the cornea [55,63].

As previously mentioned (see ‘Epidemiology’), the majority of global LSCD cases are female [5,22]. Although the reason for this remains unclear, it may be associated with a higher occurrence of dry eye attributed to hormonal regulatory factors [11,24]. Additionally, it is likely that women wear CL over a longer duration and with more frequency [11]. Further investigation into more underlying discrepancies is necessary to fully grasp the factors contributing to gender predisposition.

### 4.3. Concomitant Ocular Disease

While pre-existing ocular conditions could alone cause LSCD with long-term inflammatory damage, there have been associated risks with numerous CL-induced LSCD cases [2]. Under pre-existing pathological conditions, CL wear increases damage, resulting in cumulative inflammatory and hypoxic stress and significant LSC dysfunction [24,30]. High myopia has been identified as a risk factor for CL-induced LSCD [23], and concurrent ocular diseases associated with CL-induced LSCD include dry eye, blepharitis, pterygium, keratoconjunctivitis sicca, herpes simplex keratitis, severe meibomian gland dysfunction [24,27]. The association with rosacea is subject to debate; while some studies have shown associations, Termote et al. do not support this [22]. Ocular co-morbidity and subclinical diseases may include severe viral conjunctivitis, prior exposure to thimerosal and severe allergic conjunctivitis [24].

## 5. Diagnosis

**Figure 1:**
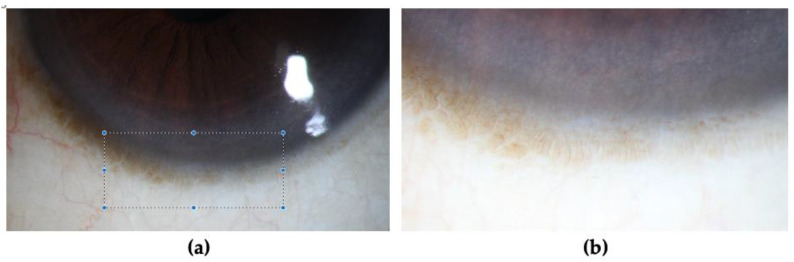
(**a**) Slit-lamp images showcasing limbal palisades of Vogt. (**b**) Zoomed-in view.

Symptomatic patients typically present with pain, tearing, redness, irritation, dryness, photophobia, decreased vision and blepharospasm [29,64]. Although the disease is usually bilateral, patients mostly present asymmetrically [65]. Diagnostic confirmation is typically achieved through clinical and slit lamp examination [64]. Hence, regular annual screening using slit lamp examination is important and should be performed for all CL wearers [12,23]. Specific evaluation of the superior and inferior limbus is crucial since they are most commonly affected in CL-induced LSCD [66,67]. Palisades of Vogt are usually absent in LSCD [23,64]; however, this cannot be confirmed on slit lamp examination as they are not always visible [68]. Nevertheless, their presence excludes the diagnosis of LSCD [8]. Figure 1 shows limbal palisades of Vogt. 

**Figure 2:**
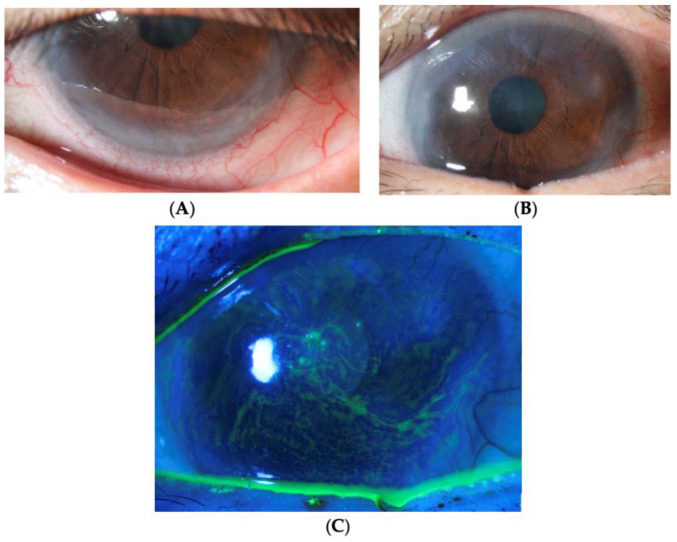
Clinical images of patient with CL-induced LSCD. (**A**,**B**) Opaque peripheral epithelium in view of invasion of conjunctival tissue with cornea vascularization and pannus formation. (**C**) Punctate staining in a curve-like path (whorled epithelium).

**Figure 3:**
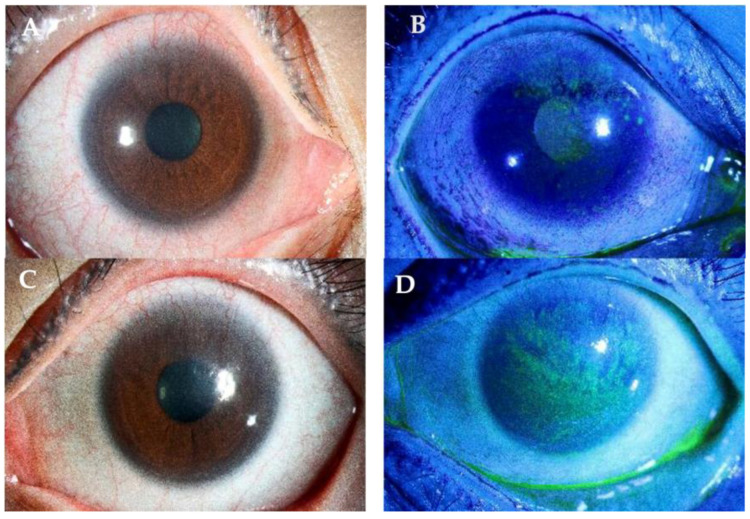
Bilateral eyes: clinical images of the same patient with CL-induced LSCD. (**A**,**B**) Right eye cornea vascularization with fluorescein staining. (**C**,**D**) Left eye cornea pannus formation, vascularization encroaching onto visual axis with late fluorescein-staining pattern.

The hallmark feature of LSCD is conjunctivalization, marked by the migration of conjunctival cells to the cornea [11,64]. The cornea becomes more opaque with conjunctival cells, leading to whorls of opaque epithelium seen upon examination as the epithelium grows in a spiral pattern (Figure 2C) [11]. As conjunctivalization progresses, a late staining pattern occurs on fluorescein staining (Figure 3D) [69]. This happens as conjunctival epitheliums have “looser” intercellular junctions than corneal epitheliums. leading to greater uptake and better permeability to fluorescein [70].

The staining fluorescein patterns and other clinical signs on slit lamp examination can further delineate the various stages of disease progression (Table 2).

Different investigative modalities have been used and assessed for sensitivity and reliability in diagnosing and staging LSCD. This includes impression cytology, in vivo confocal microscopy, histologic markers and AS-OCT.

### 5.1. Histologic Markers

To confirm conjunctivalization in the cornea, cytokeratin 7, 13, and 19 are markers specifically expressed in conjunctival epithelial cells, while mucin 5AC (MUC5AC) is used for the detection of goblet cells [73,74]. The degree of fluorescence exhibited by conjunctival and goblet cell markers also helps quantify the severity of the disease [73]. For LSCs, identifying a definitive, specific histologic marker is still an ongoing challenge that has yet to be fully solved [64]. In the corneal epithelium with various cell types, LSCs could be identified by staining characteristics. LSCs are known to stain positively for DeltaNp63, ABCG2, and ABCB5, and negatively for Cx43 and K3/K12 [75]. The presence of the cytokeratin 12 marker can then be used to quantify whether the disease is considered mild or partial [76,77]. However, other epithelial cells like transit amplifying cells also exhibit similar staining patterns with the aforementioned markers [75]. Thus, there is still room for future research to identify a marker that determines LSC status more quantitatively and specifically [8]. 

### 5.2. Impression Cytology

Impression cytology is best used in clinically non-diagnosable cases with high suspicion of LSCD [67,78]. In the corneal epithelium, impression cytology can detect mucin, which would suggest conjunctival goblet cells are present [67,69]. However, the detection of goblet cells has low sensitivity, so its absence does not exclude LSCD, and a repeat cytology would be required [64,67]. The presence of goblet cells is considered the sine qua non of LSCD [64], but their absence does not mean that the limbus is healthy [64]. Cytokeratin markers are also used to differentiate corneal (Cytokeratin K3) and conjunctival (Cytokeratin K19) epithelial cells [23,25]. Martin et al. reported the epithelial phenotype of the pannus in LSCD patients with negative staining to a cornea-specific anti-keratin monoclonal antibody (K3) and a positive presence of a conjunctival-specific anti-keratin monoclonal antibody (K19) [23]. Even though impression cytology is not routinely performed in contact lens practice, it can potentially help to evaluate subclinical disease in a fellow eye [8]. It could also have an important role in evaluating patients undergoing therapeutic penetrating keratoplasty to select individuals who would benefit from LSC transplantation [25].

### 5.3. In Vivo Confocal Microscopy

In vivo confocal microscopy (IVCM) can further assist in identifying LSCD by amplifying specific microstructural changes (goblet cells associated with conjunctivalization) not seen in slit lamp examination [71,79]. It can better assess disease severity and both confirm the diagnosis and monitor LSCD progression, albeit with several challenges [71,79]. Widespread use is limited due to cost and it being limited to academic centers [64,79]. It is also technically demanding with its difficulty of use and small field of view [64,79]. As compared to impression cytology, it has a similar detection rate but potentially lower sensitivity for detecting goblet cells due to a smaller field of view and variable goblet morphologies described in both hypo- and hyper-reflective cytoplasm [13].

### 5.4. AS-OCT

This imaging modality has shown promise in diagnosis and monitoring due to its noninvasive and comprehensive ocular surface visualization with low operator dependence [64,80]. Ladan et al. showed that AS-OCT was clinically significant in clinical evaluation and surgical planning for LSC transplantation surgery [80]. It is also important for intraoperative guidance in cultivated limbal epithelial stem cell transplantation (CLET) [81]. Its non-contact rapid scanning and pachymetry mapping help in identifying the delicate fibrovascular pannus for removal [81]. It has great diagnostic potential in identifying characteristic findings of increased epithelial thinning and absent palisades of Vogt [13, 82). Epithelial thinning, while a nonspecific finding, could confirm the presence of LSCD as the degree of thinning is different [13,82]. While most other disorders have a thinning of <10%, a 20–30% thinning has been reported in eyes with LSCD [83,84,85].

The AS-OCT can further provide volumetric scans of the limbus that give a three-dimensional image, which helps quantify LSCD severity [86,87]. Unlike IVCM, AS-OCT simplifies image acquisition for features like the palisades of Vogt with less time required and less operator dependence [13].

### 5.5. OCT-Angiography (OCT-A)

The use of OCT-A has been explored for quantifying changes seen in both limbal vascularization and corneal neovascularization [88]. As LSCD severity increases, the density of vascularization and the extent of invasion into the cornea progressively increase [89]. OCT-A can differentiate LSCD from other mimics with similar corneal vascularization [13,90]. Neovascularization in LSCD occurs in the superficial layers, whereas non-LSCD cases have neovascularization in the deep stromal layers [13,90]. Hence, a significant reduction in vascular density is noted when the superficial layer is segmented [13,90]. When the superficial vascular density values are >0.38, the diagnosis of LSCD can be confirmed with a sensitivity and specificity of 97.9% and 73.8%, respectively [13,90].

## 6. Management

### 6.1. Conservative Management

Conservative measures are always first-line treatment for symptomatic relief and the resolution of disease in mild–moderate cases. A case series by Termote et al. demonstrated successful outcomes in all 17 patients with CL-induced focal LSCD, with improved BCVA in 20 eyes (74%) and stability in 7 eyes (26%) [22]. Surgical intervention for focal LSCD was not required [22]. However, it may not be enough to fully treat severe LSCD. In a case series on CL-induced LSCD with thimerosal use, symptomatic improvement and LSCD resolution only occurred in one patient (*n* = 6, 16.7%) after stopping CL use [12], and another study showed that all severe CL-induced LSCD cases failed conservative management (*n* = 12, 100%) [24]. With the noted conflicting results from the aforementioned studies, this warrants more clinical studies with larger cohorts and a heavier emphasis on mild–moderate CL-induced LSCD. Nonetheless, conservative treatment has generally yielded successful outcomes since most CL-induced LSCD cases are clinically reversible [29,66,91]. In mild cases, a stepwise approach is recommended, starting with aggressive first-line treatment of dry eye (preservative-free artificial tears), followed by topical corticosteroids, and then with other advanced adjunct medications [10]. In severe LSCD, there should be concurrent use of dry eye medications (preservative free-artificial tears, vitamin A, autologous serum tears) and anti-inflammatory treatment (topical steroids, oral doxycycline, topical cyclosporine) [10,27]. Even in cases requiring surgery, targeted conservative therapy for dry eye and inflammation remains essential to avoid poor surgical outcomes [10,27,64].

Highly recommended for all cases, discontinuing CL wear is associated with improved ocular stability and symptomatic improvement, as seen across multiple case studies [11,12,66]. Stenson et al. documented four CL-induced LSCD cases responding to discontinuing CL use [66], while Fuerst et al. reported slow symptomatic improvement in thirteen LSCD patients, with 54% receiving additional silver nitrate treatment [26]. However, this alone might not suffice for severe cases, such as in Jenkin et al., where five of six patients failed to improve in symptoms after CL and thimerosal exposure discontinuation for up to 7 years of follow-up [12].

Treating dry eye specifically is crucial to addressing a key component of CL-induced LSCD pathophysiology. To enhance epithelial repair and improve the corneal epithelial environment, dry eye should be aggressively treated with preservation-free lubrication [12,29]. Symptom resolution within months was noted in several case series where CL-induced LSCD patients with thimerosal exposure began artificial tear use and discontinued CL use [12]. For CL-induced LSCD with blepharitis and meibomian gland dysfunction, treatment with lid hygiene/warm compresses and doxycycline was shown to be effective [12]. Recent evidence indicates oral omega-3 fatty acid supplementation could improve dry eye symptoms and tear film breakup time, helping with symptomatic improvement [92]. 

Advanced second-line treatment (topical vitamin A, autologous serum (AS) tears) should be considered when first-line treatment has failed [92]. AS has demonstrated potential for reversing severe LSCD and preventing the need for surgical intervention [7]. A case series shows symptomatic stabilization and resolution for all 20 eyes of 14 patients with severe CL-induced LSCD following an aggressive treatment regimen of autologous serum eye drops [7]. Topical vitamin A helps promote epithelial differentiation, and serum tears enhance epithelial function via its growth factors and “nutrients” [7,93]. Punctal/cautery occlusion can also be considered [7]. Retinoic acid is generally effective in treating dry eye but has not been reported to be consistently effective in CL-induced LSCD [7].

Anti-inflammatory treatment involving topical corticosteroids and cyclosporine has been shown to be beneficial [7,24,91], improving tear film and reducing conjunctival inflammation with complete symptom resolution [7,24,91]. However, in Sendele et al., antibiotics and corticosteroids were discontinued when clinical outcomes for patients remained unaffected [7,24,91].

Since the causal agent is related to the patient’s current CL, changing CL type and/or CL care helps with resolving symptoms [7,24,91]. Patients with refractive error should switch to wearing glasses instead [7,24,91]. Implementing activity- and CL-specific modifications, such as fitting, CL type and reduced usage time, supports a more effective transition back into CL use [66]. Patients should also consider changing their cleaning solutions to preservative-free or heat-based types [66]. With complete resolution and elimination of the offending agent, soft CL use may then be restarted [8]. However, Termote et al. showed that seven out of seventeen patients still experienced pain and redness despite a new CL type and were unable to resume CL wear [22]. Disposable silicone hydrogel and rigid gas permeable lenses have been utilized for refractive error after CL-induced LSCD [22,26]. Some patients were successfully treated with scleral/PROSE lenses [22]. Rossen et al. noted good therapeutic results with scleral/PROSE lenses in 10 patients, even though these clinical data are unpublished [8]. It is advised that CL use should be discontinued at any signs of disease recurrence [8]. Some patients have undergone LASIK after previous CL-induced LSCD treatment [23].

### 6.2. Surgical Management

However, some severe cases will persist despite conservative treatment. This is when a variety of surgical options are considered for definitive management. Conservative management should still be applied as an adjunct to continue nourishing the limbal niche [8]. In CL-induced LSCD, surgical considerations will differ from just treating LSCD in general due to its specific features.

#### 6.2.1. Superficial Keratectomy (SK)

SK, a form of mechanical debridement, has consistently shown efficacy as a monotherapy for CL-induced LSCD [11,72]. It may have immediate symptomatic improvement, but recurrence is frequent [11,72]. In certain instances, it has been useful with other treatment modalities, like lubrication, topical steroids or alternative surgical options [11,65,72,94]. In aggressive LSCD cases where the regenerative potential of LSCs is severely impaired, debridement of the conjunctival epithelium alone is insufficient to cure CL-induced LSCD [11,72]. For a patient in Jeng et al.’s study, the conjunctivalized epithelium persisted on the corneal surface despite two initial epithelial debridements, eventually requiring more aggressive surgery [11,72].

#### 6.2.2. Phototherapeutic Keratectomy (PTK)

PTK is usually employed to treat superficial stromal scarring that develops with LSCD. Rossen et al. suggest that PTK is most effective after LSCD has been treated, as stromal scarring is more likely to regress once a stable corneal epithelium has been restored [8].

#### 6.2.3. Penetrating Keratoplasty (PK)

PK has been noted as an effective monotherapy for LSCD [8]. According to Chan CC et al., primary PK should be avoided in patients with underlying ocular disorders due to the high likelihood of failure and its failure to address the underlying condition of LSCD [24]. On its own, it is only a temporalizing measure for symptomatic improvement and prevention of conjunctivalization since the transplanted graft lacks LSCs [24]. This results in an increased risk of failure and disease recurrence [24]. A study showed that the donor epithelium survives for approximately 1 year post-PK, which matches the duration of donor cornea viability before decompensation [95]. Nevertheless, it is more effective when used in conjunction with other conservative/surgical treatments that directly improve the limbal niche. Studies have demonstrated successful treatment of CL-induced LSCD through PK with other adjunct treatments like corticosteroids [36] and limbal transplantation [42,96,97]. In cases of significant corneal scarring caused by CL-induced LSCD, deep anterior laellar keratoplasty is the preferred option for corneal scar remodeling and effective visual outcomes [98]. It should be performed after LSCD has resolved and after or simultaneously with limbal transplantation [98].

#### 6.2.4. Amniotic Membrane Transplantation (AMT)

Frequently employed for the treatment of severe LSCD, AMT acts as an environment for nourishment, stimulating LSC growth and harboring anti-inflammatory properties that subsequently replenish the limbal niche [69,94]. It has been successful in treating CL-induced LSCD by providing long-term stability of the corneal epithelium [94]. However, the transplanted AM lacks any LSCs, which may result in patients’ eyes having insufficient native LSCs [99]. In such cases, the transplanted AM is unable to prevent corneal conjunctival epithelization because the migration of conjunctival epithelial cells occurs faster than the regeneration of corneal epithelial cells without properly functioning LSCs [99]. Therefore, the presence of at least a partially viable LSC source is critical for AMT success, particularly in severe or complete LSCD cases [69,94]. Based on disease severity, AMT can be implemented alone for milder cases or with limbal transplantations to treat severe to complete LSCD [64,69,94]. Compared to allogeneic limbal transplantation, it has a reduced risk of rejection and avoids complications of the donor eye or systemic immunosuppression [69,94]. Notably, in one case, a patient with bilateral diffuse LSCD opted for AMT instead of an allogeneic limbal transplant due to patient preference, achieving success in restoring and maintaining long-term corneal epithelial surface stability [11]. Anderson et al. reported clinical success in VA improvement with superficial keratectomy followed by an AMT overlay graft (*n* = 14, 92.9%) [94,100]. Furthermore, AMT has been used in conjunction with keratolimbal allografts followed by penetrating keratoplasty [94,100].

In 2017, Dua et al. introduced a procedure termed amnion-assisted conjunctival epithelial redirection (ACER) [101]. This surgical technique involves an inner AM layer that is implanted and aids corneal epithelial regeneration, and an outer AM layer placed to prevent the migration of conjunctival epithelium onto the cornea [101]. This addresses the post-operative challenge of conjunctival epithelization often encountered by AMT in severe LSCD [101]. In partial LSCD, Sang et al. demonstrated successful corneal re-epithelialization in all cases with one patient secondary to CL wear with ACER coupled with superficial keratectomy (*n* = 3, 100%) [99]. Out of ten patients with severe LSCD, Dua et al. had 80% of patients achieve complete/partial success following ACER, including one patient with CL-induced LSCD [101]. ACER holds the potential to be an effective ocular surface reconstruction technique that negates the need for LSC transplantation and the risks of immunosuppression [99].

#### 6.2.5. Autologous Limbal Stem Cell Transplantation

This option involves harvesting LSCs from the healthy donor’s eye and transplanting them into the affected eye [102]. This has been reported with good visual outcomes, significant visual acuity improvement following transplantation (*n* = 2, 100%) [11] and one successful case performed in conjunction with superficial keratectomy combined with conjunctival autograft (*n* = 1, 100%) [11]. A retrospective study showed long-term ocular stability without LSCD recurrence (late staining, neovascularization, conjunctivalization) at the last follow-up in conjunctival limbal autograft (CLAU) (*n* = 26, 100%) and in combined keratolimbal allograft/CLAU (*n* = 18, 100%) [103]. In a systematic review, Shanbhag et al. note anatomical and functional success rates of 69% and 60% in unilateral LSCD due to ocular burns [104]. Baradaran-rafii et al. evaluated CLAU surgical outcomes in 34 unilateral LSCD cases due to chemical injury, noting anatomical success (88%), visual success (88%) and graft failure (11.8%) [105]. It benefits from obviating the need for systemic immunosuppression and hence avoiding the risk of graft rejection [11]. However, a lack of autologous transplantation success can occur due to similar LSCD processes occurring in the healthy donor eye [65]. Studies have shown that a seemingly normal donor eye may have subclinical LSCD [65].

A conjunctival keratolimbal autograft requires at least a third of the donor’s healthy eye [65]. Depending on the extent of LSCD, the supposedly healthy donor eye could be jeopardized with a significant portion of the limbal source extracted for grafting, leading to clinical LSCD in the healthy eye [65]. As such, autologous limbal transplantation has shown elevated complication rates for CL-induced LSCD, including conjunctivalization (*n* = 4, 50%) [65] and donor eye complications (*n* = 5, 40%) [12] (*n* = 6, 16.7%) [97]. Therefore, an autograft is not recommended for patients with a history of CL wear and existing unilateral severe LSCD [65]. A solution to this is an ex vivo cultivated autologous limbal transplant, where a significantly smaller amount of donor tissue is required [106]. Progress in standardizing the culturing process has been made with Holoclar^®^ (Chiesi Farmaceutici SpA, Parma, Italy), the first commercial stem cell therapy approved in the EU indicated for treating moderate-to-severe LSCD secondary to ocular burns [107]. Two retrospective studies showed successful treatment outcomes with corneal stability 1 year post-Holoclar implantation (*n* = 104, 72.1%) [108]. Marchini et al. report complete epithelial restoration and ocular stability with autologous ex vivo cultured limbal stem cell grafts in LSCD cases due to chemical burns (*n* = 16, 62.6%): three cases had moderate success (18.7%), while three patients experienced graft failure (18.7%) [106].

Alternative approaches to autologous transplantation, such as ipsilateral limbal grafting from inferior to superior limbus [109], simple limbal epithelial transplantation (SLET) and narrow-strip conjunctival autografts, offer options with smaller grafts that pose less risk to the health of the remaining limbus [8].

#### 6.2.6. Narrow Strip Conjunctival Autograft

In Dupp et al., a patient with bilateral focal LSCD declined allogeneic transplantation and opted for autologous transplantation due to the refusal of systemic immunosuppression [110]. Due to this, a free conjunctival autograft from the inferior bulbar conjunctiva was utilized, fashioned after a “narrow-strip conjunctival autograft” that was previously used for treating pterygium [110]. Despite some conjunctivalized epithelium growth onto the cornea, clinical outcomes were largely successful in restoring vision and preventing the progression of conjunctivalized epithelium from obscuring the pupil entrance (*n* = 19, 94.7%) [110]. 

#### 6.2.7. Simple Limbal Epithelial Transplantation (SLET)

In SLET, a 2 × 2 mm piece of donor limbal tissue from the healthy fellow eye is divided into 8–10 small pieces, which are then distributed and affixed onto an amniotic membrane that is placed onto the cornea [111]. This minimizes donor tissue usage, which then optimizes AMT-based treatment for donor eyes with limited stem cell reserves [111]. However, in cases of severe bilateral LSCD, autologous SLET (auto-SLET) is a nonviable option, leading to the application of allogeneic SLET (allo-SLET) in conditions like Stevens–Johnson syndrome and mucous membrane pemphigoid [112].

#### 6.2.8. Living-Related Conjunctival Limbal Allograft (Lr-CLAL)

With living-related allografts, there is a lower antigenic challenge with histocompatibility matching between the donor and recipient [111]. However, implantable graft coverage is significantly limited to approximately 50% of the recipient limbus due to the limited quantity of tissue and potential donor risk [111]. Compared to cadaver-donor keratolimbal allograft (Cd-KLAL), Lr-CLAL may be more beneficial by providing healthy donor conjunctiva with goblet cells [111], but more research needs to be conducted to garner a better assessment of the long-term outcomes of both Lr-CLAL and Cd-KLAL.

#### 6.2.9. Cadaver Donor Keratolimbal Allograft (Cd-KLAL)

Cd-KLAL is the ideal option for bilateral severe LSCD that have healthy conjunctiva, and is particularly well-suited for conditions like CL-induced LSCD, iatrogenic LSCD and aniridia, which have minimally affected conjunctiva [113]. Due to the absence of healthy conjunctiva in Cd-KLAL, it is often combined with other procedures like Ir-CLAL to enhance ocular surface repair [114]. With Cd-KLAL, there would be no concern of conversely causing LSCD in the donor’s eye [111]. Histocompatibility matching is not typically performed due to impracticalities in the eye bank that supplies the donor tissue [111]. Proper long-term immunosuppressive therapy is therefore crucial for all allogeneic transplantations [111]. Careful management with an organ transplantation specialist has been shown to minimize adverse effects, toxicity and poor outcomes [65,115,116].

A case series reported a 30% (3/10) rejection rate for cadaveric allogeneic limbal grafts, with two cases responding to oral cyclosporine [96]. Apart from acute or chronic rejections, other prevalent complications include ocular hypertension and complications of chronic immunosuppression [65,116]. In cases of insufficient systemic immunosuppression, long-term reports of allo-LSC transplantation have shown attrition of the allogeneic graft survival, ranging from 21.1% to 33.3% after 4 years of follow-up [100,117,118]. Increased IOP, a common post-LSC transplantation complication, has reported rates of 33.1%, 37.2% and 37.5% [118,119,120]. Cataract formation was also noted as a complication secondary to topical immunosuppression [96]. Although systemic immunosuppression is relatively safe, it can cause persistent epithelial defects and an increased risk of infective keratitis, specifically fungal [1,111]. Certain ocular co-morbidities have been associated with poorer outcomes following LSC transplantation; this includes severe dry eye, keratinization, eyelid scarring, symblepharon, lagophthalmos and inflammatory conditions [117,120,121].

#### 6.2.10. Cultivated Limbal Epithelial Transplantation (CLET)

With CLET, the cell source could be extracted from any type of donor (patient, living relative, cadaver) and with less quantity, reducing the need for donor tissue and shortening epithelization time post-transplantation [122]. The lack of immune factors in the transplanted cells would also decrease the risk of an autoimmune reaction [122]. However, the cultivation of stem cells is mainly hindered by time and cost constraints associated with specialized facilities [123,124]. A possible concern clinicians may face is the large variability in cell source, culture technique, immunosuppressive therapy and inclusion criteria. However, Baylis et al. found no significant difference in the clinical outcomes of cultivated limbal epithelial transplantation in spite of clinical variability [125]. A 77% overall success rate was reported for cultivated allogeneic transplantation [125]. Allogeneic CLET was able to achieve a success rate of 71.4% for bilateral and total LSCD in a study by Basu et al. [122].

For bilateral LSCD, oral mucosal epithelium and conjunctival epithelium have been shown to be effective alternate non-LSC-containing stem cell sources for LSC cultivation. In a case series, six patients with LSCD secondary to chemical burns and SJS had significant and successful symptomatic improvement at 1 month (*n* = 6, 66.7%) and 1 year post-op (*n* = 6, 100%) after treatment with cultivated oral mucosal epithelial transplantation (COMET) [126].

## 7. Current Gaps

As of now, there remains a significant gap in clinical research on CL-induced LSCD. Unlike LSCD from other causes, randomized controlled trials are lacking in establishing treatment guidelines for CL-induced LSCD. This gap is further exacerbated by the limited research testing of various treatment modalities and proposed algorithms. An underestimation of subclinical cases also underscores the necessity for determining the true prevalence of the disease. Standardizing severity grading and developing a comprehensive treatment guideline are imperative to enhance our understanding, identification and management of CL-induced LSCD.

## 8. Conclusions

CL-induced LSCD is a rarer subtype of LSCD with unique pathogenesis and management considerations. Nonetheless, good CL hygiene cannot be further stressed to prevent CL-related complications, including the type and duration of CL worn. To best minimize disease progression, patient education and routine, regular slit-lamp examination screenings for soft CL users are vital. This will help early identification of the disease with conservative treatment like discontinuing/modifying CL wear, dry eye treatment and anti-inflammatory treatment, potentially averting the need for surgery. Surgical options would be AMT and LSC transplantation and adjunctive methods to improve visual outcomes.

Many advancements have been made with investigative modalities, surgical approaches, novel techniques and potential non-limbal stem cell sources. While there is sufficient understanding for managing CL-induced LSCD, its complex pathogenesis warrants further research. The precise mechanism and explanation for why certain CL users manifest CL-induced LSCD while others do not remain uncertain. In bilateral severe CL-induced LSCD, an allogeneic transplant may not suffice for some patients where long-term immunosuppression is not well tolerated. Our team postulates that refractive CL with drug-eluting abilities may decrease the incidence of CL-induced LSCD. There has been research on CL with drug-eluting abilities, and combining it with refractive purposes may help slow down disease progression, especially in patients who are insistent on still using CL. In the future, avenues for research will hopefully address these unknown gaps and concerns.

## Figures and Tables

**Table 1:** Etiologies of Limbal Stem Cell Deficiency [8,10,11,12,13,14].

Congenital	- Congenital aniridia - Multiple endocrine deficiency - Ectodermal dysplasia - Epidermolysis bullosa - Xeroderma pigmentosum - Coloboma
Traumatic/acquired	- Ocular burns (chemical/thermal) - Post-surgical - Contact lens wear - Radiation - Drug-induced - Peripheral ulcerative corneal diseases - Neurotrophic keratopathy - Neoplasia - Multiple hormonal deficiencies
Autoimmune	- Stevens–Johnson syndrome - Toxic epidermal necrolysis - Mucous membrane pemphigoid - Sjogren’s syndrome (primary and secondary) - Vernal keratoconjunctivitis - Graft vs. host disease
Idiopathic	

**Table 2:** Fluorescein Staining Patterns in LSCD.

Early	Fluorescein staining in a punctate pattern in a curve-like distribution: Commonly present in the superior cornea [10,71].
Late	Punctate staining merges into a linear pattern.Eventually progresses into a centrally distributed confluent sheet [10,37].
Later-stage	Corneal pannus, which loosely refers to [10,28]: Sheet of conjunctival-type epithelium; Neovascularization; ○ Superficial neovascularization develops first, ultimately leading to corneal neovascularization; ○ Often spreads from peripheral to central.
End-stage	Signs include [71,72]: Ulceration, perforation (due to recurrent epithelial defects); Corneal scarring; Near total vision loss.

## Data Availability

No new data were created or analyzed in this study. Data sharing is not applicable to this article. There are no supplementary data available for this article.
